# Rising Influence of Nanotechnology in Addressing Oxidative Stress-Related Liver Disorders

**DOI:** 10.3390/antiox12071405

**Published:** 2023-07-09

**Authors:** Sathiyamoorthy Padmanaban, Durgasruthi Pully, Antony V. Samrot, Vijayakumar Gosu, Nanthini Sadasivam, In-Kyu Park, Kamalakannan Radhakrishnan, Don-Kyu Kim

**Affiliations:** 1 Department of Biomedical Sciences and BioMedical Sciences Graduate Program (BMSGP), Chonnam National University Medical School, Gwangju 61469, Republic of Korea; 207904@jnu.ac.kr; 2 Biochemistry and Biotechnology, Faculty of Science, KU Leuven, 3000 Leuven, Belgium; durgasruthi.pully@student.kuleuven.be; 3 School of Bioscience, Faculty of Medicine, Bioscience and Nursing, MAHSA University, Jenjarom 42610, Malaysia; antony.s@mahsa.edu.my; 4 Department of Animal Biotechnology, Jeonbuk National University, Jeonju 54896, Republic of Korea; gosu@jbnu.ac.kr; 5 Department of Integrative Food, Bioscience and Biotechnology, Chonnam National University, Gwangju 61186, Republic of Korea; 218545@jnu.ac.kr; 6 School of Biological Sciences and Technology, Chonnam National University, Gwangju 61186, Republic of Korea

**Keywords:** antioxidants, oxidative stress, ROS, nanoparticles, liver disease

## Abstract

Reactive oxygen species (ROS) play a significant role in the survival and decline of various biological systems. In liver-related metabolic disorders such as steatohepatitis, ROS can act as both a cause and a consequence. Alcoholic steatohepatitis (ASH) and non-alcoholic steatohepatitis (NASH) are two distinct types of steatohepatitis. Recently, there has been growing interest in using medications that target ROS formation and reduce ROS levels as a therapeutic approach for oxidative stress-related liver disorders. Mammalian systems have developed various antioxidant defenses to protect against excessive ROS generation. These defenses modulate ROS through a series of reactions, limiting their potential impact. However, as the condition worsens, exogenous antioxidants become necessary to control ROS levels. Nanotechnology has emerged as a promising avenue, utilizing nanocomplex systems as efficient nano-antioxidants. These systems demonstrate enhanced delivery of antioxidants to the target site, minimizing leakage and improving targeting accuracy. Therefore, it is essential to explore the evolving field of nanotechnology as an effective means to lower ROS levels and establish efficient therapeutic interventions for oxidative stress-related liver disorders.

## 1. Introduction

Reactive oxygen species (ROS), which have a role in cellular signaling, are produced naturally as byproducts of regular cell activity. The dysfunction, structures, and homeostasis of cells are adversely affected by an increase in ROS levels [1]. This brings about oxidative stress. As a result, altering cellular redox equilibrium increases the chance of developing several illnesses [2]. The most frequent ways for oxygen to be reduced involve stepwise transfers of one electron, which creates free radicals such as the superoxide anion (O_2_^•−^ radical) and hydroxyl radical (^•^OH radical), or non-radical oxidant species such as hydrogen peroxide (H_2_O_2_). Among these, hydrogen peroxide is found to be the most abundant in eukaryotic cells [3]. ROS will be usually high during respiration and photosynthesis due to the transfer of electrons to oxygen via the electron transport system [4,5]. Numerous metabolic processes, mostly those that take place in the mitochondria, produce intrinsic ROS. The development of living species depends on these reactions. Oxidative stress brought on by ROS overexpression can result in the breakdown of biological components [6]. Excessive ROS production causes oxidative stress, which is linked to a variety of diseases such as cancer, neurological disorders, inflammation, etc. [7,8]. ROS has two sides to it. A moderate amount of ROS can serve as a second messenger for physiological control. However, an excessive amount of ROS can overwhelm a cell’s antioxidant defenses and lead to cell death [9,10]. Endogenous biological ROS generators include NOXs, hypoxia, metabolic defects, ER stress, lipoxygenases, and redox-cycling metals such as Fe and Cu [11,12,13,14,15,16,17]. Maintaining the equilibrium between ROS generation and clearance ensures cellular redox homeostasis [18]. In contrast, intracellular antioxidants such as superoxide dismutase (SOD), catalase (CAT), nuclear factor erythroid 2-related factor 2 (NRF2), glutathione (GSH), NADPH, and many dietary antioxidant compounds (e.g., vitamin C, vitamin E, selenium, etc.) can scavenge the endogenous biological ROS generators [19,20,21,22,23,24]. Maintaining the redox equilibrium is crucial for preserving normal physiological processes and lowering the prevalence of illnesses. Numerous clinical dysfunctions, including aging and cancer, will take place if the imbalanced redox status exceeds the cellular tolerance thresholds. 

Nanoparticles are referred to as materials with a total dimension between 1 and 100 nanometers [25,26,27,28]. The unique characteristics of nanoparticles, such as their size, chemical reactivity, energy absorption, biological mobility, strength, surface area, sensitivity, and stability, distinguish them from bulk materials. Nanoparticles have become significant components in modern medicine, functioning as gene carriers for precise cell distribution and as contrast agents in medical imaging [29]. Nanomaterials can be categorized into metallic and non-metallic nanoparticles based on their constituent materials. Metallic nanoparticles (MNPs) refer to a type of nanoparticle that is composed of inorganic metals, metal oxide cores, or metal-associated particles. Several metallic nanoparticles are widely recognized, including aluminum [30], gold [31], iron [32], copper [33], silver [34], cerium [35], manganese [36], zinc [37], titanium oxide [38], nickel [39], quantum dots [40], and others. The category of non-metallic nanoparticles encompasses various types of nanoparticles, such as ceramic-based, carbon-based, silica-based, and biological macromolecule-derived nanoparticles [41,42]. Nanoparticles are utilized in diverse applications, such as the remediation of wastewater, specifically in the elimination of heavy metals. They are also employed as antibacterial, antioxidant, and anticancer agents. Additionally, they are employed in drug delivery and self-oxidation. ROS increase can be a fatal disorder, but our system has an antioxidant system to combat the alterations in the ROS levels [43,44,45,46]. Usually, ROS levels will be high at tumor sites, inflammatory regions and where the antioxidant system has failed to manifest its ability. Upon rapid increase in the ROS external antioxidants can be supplied as a supplement—antioxidants such as vitamin E, vitamin C, vitamin A, lycopene, lutein, and more. After the advent of nanotechnology, a variety of ROS-scavenging substances have been produced with enzyme-mimicking properties, such as SOD/CAT-like activities. This advancement of enzymology aims to reduce aberrant ROS levels to levels that are consistent with cellular biological function. The use of nanoparticles in the clinical field has increased exponentially after the outbreak of COVID-19. In this review, we will look at different nanoparticles’ antioxidant effects and how liver research might benefit from them.

## 2. Oxidative Stress—A Booming Target

Oxidative stress is a result of an imbalance between the restricted antioxidant defenses and the excessive production of reactive oxygen/nitrogen species (ROS/RNS) [47,48]. An immediate effect of excessive ROS generation is the modification of cellular biomolecules such as DNA, lipids, and proteins, which can result in cell death. ROS are produced endogenously as byproducts of a variety of enzymatic reactions and metabolic pathways that require molecular oxygen [48]. These ROS are likely involved in the pathogenesis of various human diseases, including liver diseases such as alcoholic liver disease (ALD), non-alcoholic fatty liver disease (NAFLD), drug-induced liver injury (DILI), hepatitis, cirrhosis, Wilson’s disease, hemochromatosis, non-alcoholic steatohepatitis (NASH), and primary biliary cholangitis (PBC) [49,50]. Exogenous sources of ROS include ionizing radiation, diet, metals, pesticides, or other toxic compounds [49,51,52]. ROS comprise a variety of molecular entities that are derived from oxygen. Among them, the most notable is the superoxide (O_2_^•−^) anion, which is a prototypical ROS generated through enzymatic processes and in the electron transport chain (ETC) of mitochondria [53]. Moreover, other oxidants, for example hydrogen peroxide (H_2_O_2_) or hydroxyl radicals (^•^OH), are also classified as ROS. The majority of ROS are generated in mitochondria, where superoxide anion is a generated as a by-product of the transfer of electrons to oxygen in the respiratory chain, leading to the production of adenosine triphosphate (ATP) [54]. 

Despite its role in macromolecular damage, the involvement of ROS in the etiology of various diseases is not limited. There is an ever-growing body of evidence which suggests that ROS signaling plays a role in the etiology of diseases. Oxidative stress has been linked to carcinogenesis, neurodegenerative diseases, atherosclerosis, diabetes, aging, and haptic pathogenesis due to the direct or indirect impact of ROS on nucleic acids, proteins, and lipids [55,56,57]. ROS has been demonstrated to stimulate tumor metastasis via the upregulation of gene expression, as evidenced by Klaunig JE and Wang Z [58,59]. Cellular oxidants have been implicated in the activation of transcription factors. The abundance of evidence supporting the involvement of ROS in modulating cellular signaling pathways implies that the pressing question is to determine the mechanism by which ROS initiate cellular signaling [60]. The oxidative interface is a conceptual region which reflects the interaction between ROS and the signaling molecules, which they induce to activate oxidative stress-responsive pathways [60,61]. There have been notable implications of oxidants on signaling pathways, particularly on the MAP kinase/AP-1 and NF-κB pathways. The activation of these transcription factors has been implicated in both cellular proliferation and the process of apoptosis. The concentration of ROS in cells appears to be a factor in the selective activation of transcription factors and may contribute to the understanding of how exposure to ROS can lead to either cell death or cell proliferation [61]. 

Liver diseases pose a significant global medical challenge due to the liver’s crucial role as the primary detoxifying organ and regulator of metabolic homeostasis. The liver is responsible for the metabolism of diverse compounds that generate ROS [62,63]. Hepatic prooxidants refer to ROS that have the potential to induce liver tissue damage. The levels of hepatic prooxidants may be elevated due to various factors such as drug intake, infection, external exposures, and tissue injury, among others. Oxidative stress may arise due to an escalation in the formation of prooxidants or a deficiency in antioxidants [64,65]. The liver system is regulated by various bias-involved signaling mechanisms. These mechanisms maintain a balance of redox through molecular redox switches, oxygen sensing by the thiol redox proteome, and NAD/NADP and phosphorylation/dephosphorylation systems [64]. Understanding all these underlying mechanisms in ROS generation and effect of ROS in shifting the natural flow of metabolism could be a promising target for therapeutic outcomes of liver diseases.

## 3. ROS—A Hanging Blade in Liver Disorders

The utilization of ROS can have both beneficial and detrimental effects. An overabundance of ROS can impede numerous fundamental cellular processes. Various cellular components, including the mitochondria, peroxisomes, and endoplasmic reticulum (ER), produce ROS as part of their normal physiological functions. Mitochondria are widely recognized as the primary source of ROS in most cells, particularly in relation to ROS generated through energetic metabolism [66,67,68]. Usually in liver, hepatocytes possess robust non-enzymatic and enzymatic antioxidant defense mechanisms that effectively counteract the deleterious effects of free radicals. The superoxide dismutase (SOD) enzyme serves as the primary defense mechanism in mitigating the formation of superoxide radicals within the ETC by converting them into hydrogen peroxide (H_2_O_2_) and oxygen (O_2_). The enzyme catalase (CAT) plays a significant role in the detoxification of H_2_O_2_. The expression of CAT in mitochondria is absent due to its predominant localization in peroxisomes. Consequently, alternative antioxidant enzymes are responsible for the degradation of mitochondrial hydrogen peroxide. The reduction of H_2_O_2_ is catalyzed by mitochondrial glutathione peroxidases (Gpx1,4) and various hydroperoxides (PrxIII, Trx2) through a chain reaction that utilizes GSH as the electron donor. This process is followed by the conversion of GSH disulfide (GSSG) back to GSH, which is facilitated by the NADPH-dependent glutathione reductase (GR). Therefore, it can be inferred that the presence of mitochondrial GSH (mGSH) plays a significant role in safeguarding the mitochondria against the absence of CAT, indicating that its accessibility is essential for maintaining appropriate redox levels within the liver cells. 

ROS are highly reactive molecules that play a significant role in the pathogenesis of various liver disorders, including hepatic steatosis. ROS can induce oxidative stress in liver cells, leading to lipid peroxidation and the subsequent accumulation of fat in the liver, resulting in hepatic steatosis [69]. Among other hepatic diseases, hepatic steatosis stands out as an important condition that demands great attention due to several key reasons. First and foremost, hepatic steatosis is highly prevalent and is considered the most common liver disorder worldwide. Its prevalence has been steadily increasing in parallel with the global obesity epidemic [70]. Furthermore, hepatic steatosis is a significant risk factor for the development of more severe diseases, such as NAFLD and NASH. NAFLD can progress to NASH, which is characterized by liver inflammation, fibrosis, and can ultimately lead to cirrhosis, liver failure, and hepatocellular carcinoma. Moreover, hepatic steatosis is closely associated with metabolic disorders such as obesity, insulin resistance, type 2 diabetes, and cardiovascular disease [71]. It is considered hepatic manifestation of metabolic syndrome, a cluster condition that increases the risk of heart disease, stroke, and other health problems. Addressing hepatic steatosis is crucial not only for liver health but also for overall metabolic health and reducing the risk of associated complications. Lastly, hepatic steatosis is potentially reversible in its early stages, highlighting the importance of early detection and intervention [72]. Addressing hepatic steatosis requires a comprehensive approach that focuses on reducing oxidative stress, promoting antioxidant defense and targeting the underlying metabolic abnormalities to prevent the progression of more advanced liver diseases. Overall, hepatic steatosis is an important condition to be addressed among other hepatic diseases due to its high prevalence, associated with more severe liver disorders, its close link with metabolic abnormalities, and its potential reversibility in the early stages.

### 3.1. Role of Oxidative Stress in Hepatic Steatosis

Oxidative stress plays a significant role in the development and progression of hepatic steatosis. It occurs when there is an imbalance between the production of ROS and the body’s antioxidant defense mechanisms. In the context of hepatic steatosis, excess accumulation of fat in the liver cells leads to increased oxidative stress [70]. One of the primary mechanisms through which oxidative stress contributes to hepatic steatosis is lipid peroxidation. ROS can attack and oxidize lipids present in the liver cells, leading to the production of lipid peroxides. These peroxides can cause damage to cell membranes, disturb cellular functions, and further perpetuate oxidative stress [69]. Moreover, the breakdown products of lipid peroxidation can activate inflammatory pathways and promote liver inflammation, contributing to the progression of hepatic steatosis. Oxidative stress in hepatic steatosis also affects mitochondrial function. Mitochondria, the cellular powerhouses responsible for energy production, are highly vulnerable to oxidative damage. ROS can impair mitochondrial function, leading to reduced energy production and increased lipid accumulation in the liver cells [73,74]. Furthermore, oxidative stress-induced inflammation plays a crucial role in hepatic steatosis. Increased ROS production triggers the activation of inflammatory pathways and the recruitment of immune cells to the liver [70]. Chronic inflammation contributes to liver injury, fibrosis, and the progression of hepatic steatosis into NASH.

In NAFLD and NASH, oxidative stress encourages the activation enzymatic and non-enzymatic antioxidant mechanisms that work to reduce the ROS generation [75]. This is an additional indirect proof of the crucial part oxidative stress plays in the pathogenesis of the illness. In fact, both clinical and experimental research demonstrate that these antioxidant pathways are altered during the development of NAFLD [75]. In fact, individuals with NAFLD have higher levels of SOD and GPX activity. In vitro, pro-fibrotic and pro-inflammatory genes are expressed more frequently by HSC lacking the GPX7 isoform in reaction to FFA exposure [76]. Overexpression of GPX7 in these cells reduces ROS production and the expression of pro-fibrotic and pro-inflammatory genes, which is consistent with these findings. Choline-deficient, L-amino-defined, high-fat diet (HFD)-induced NASH fibrosis is made worse in vivo by GPX7 loss [77]. Paraoxonase-1 is an antioxidant enzyme found in the liver which can hydrolyze peroxide and lactones that are linked to lipoproteins. The study observed a group of 81 patients diagnosed with NAFLD and found a reduction in serum paraoxonase-1 concentration. This decrease may indicate a higher level of oxidative stress in these patients [78]. The upregulation of glutaminase 1 (GLS1) has been observed in preclinical mouse models of NASH, as well as in liver biopsies obtained from patients diagnosed with clinical NASH. The promotion of glutamine fueling of anaplerotic mitochondrial metabolism by GLS1 leads to an elevation in ROS generation. The inhibition of GLS1 in mice fed with a methionine/choline-deficient diet (MCD) results in a reduction of hepatic triglyceride accumulation by restoring the export of very low-density lipoprotein (VLDL) and a decrease in oxidative stress (OS) through the reduction of ROS production. The present model exhibits a correlation between GLS1 insufficiency and a reduction in lipid peroxidation.

### 3.2. Regulation of Lipid Metabolism in Hepatic Steatosis

In the context of oxidative stress, ROS such as singlet oxygen, superoxide anion, H_2_O_2_, and hydroxyl radical are the most significant oxidizing factors. These ROS are generated because of perturbations in the electronic flow along the respiratory chain, followed by the interaction of intermediates with oxygen. ROS production occurs even under normal physiological conditions. A small but notable proportion of the overall oxygen consumption, ranging from 0.15% to 5%, is allocated towards the generation of superoxide anions. Nonetheless, this reactive species is effectively sequestered by the antioxidant mechanisms present within the cell, such as catalase, SOD, and GSH [79,80]. The toxicity of ROS is attributed to their capacity to swiftly interact with multiple cellular structures. Initially, the interaction occurs between the lipids present in liver cells, leading to the generation of aldehydes, specifically 4-hydroxynonenal (HNE) and malondialdehyde (MDA). Both exhibit a prolonged half-life in comparison to ROS and possess the capability to inflict harm upon cellular structures that are situated at greater distances. In greater depth, HNE and MDA have the capability to primarily target polyunsaturated fatty acids (PUFA) [80,81]. The presence of significant quantities of PUFA in mitochondrial membranes is essential for the appropriate formation of respiratory complexes. However, the generation of aldehydes at the final stage of this process poses a threat to cellular respiration. Both HNE and MDA have the capability to enhance the proteolysis of apolipoprotein B, which results in a decrease in the secretion of VLDL and the exacerbation of pre-existing liver steatosis [80]. 

An additional significant discovery pertains to the capacity of ROS to directly target the mitochondrial DNA of the liver. The impairment, which results in a diminished production of polypeptides that make up the respiratory chain, was verified through the decrease in mitochondrial DNA levels in the hepatocytes impacted by NASH [82]. TNF-α can also stimulate ROS production in identical patients, which results in the retention of electrons along cytochrome b and the consequent impairment of the hepatic mitochondrial respiratory chain [83].

The production of ROS in the liver is also associated with the potential difference across the mitochondrial membrane (ΔΨ). Hyperpolarization of the mitochondrial membrane results in a deceleration of electron transport, promoting the association of electrons with oxygen and, consequently, the generation of ROS. The phenomenon can be restricted by the existence of an uncoupling agent which facilitates the re-entry of protons into the mitochondrial matrix. This phenomenon diminishes the disparity in membrane potential and, consequently, the generation of ROS. The redox state of cells has the potential to impact the functioning of various enzymes that play a role in lipid metabolism. These enzymes are responsible for carrying out post-translational modifications such as glutathionylation and carbonylation. Additionally, they can act as second messengers or bring about conformational changes in nuclear receptors by modulating phosphatases/kinases [84,85]. 

The establishment of the intracellular redox state is attributed to various redox pairs, including oxidized/reduced nicotinamide adenine dinucleotide (NAD+/NADH) and oxidized/reduced GSH [86,87]. The ratios function as an indicator of the presence of reducing equivalents necessary for the process of lipogenesis. The study reveals that in the context of steatotic liver, there is an elevation in lipid oxidation and tricarboxylic acid cycle, while ketogenesis remains unaltered. This observation suggests that hepatocytes engage in a compensatory mechanism aimed at mitigating the effects of lipid excess by augmenting the oxidation process. Excess fatty acids must undergo degradation to prevent lipotoxicity. Generally, the breakdown of fatty acids and their esters predominantly occurs through a cellular process known as β–oxidation. It is a metabolic pathway that occurs specifically in the liver cells to sequentially break down the long chain fatty acids into acetyl-CoA units. With further elaboration, the process of liver β-oxidation comprises four distinct reactions that produce reducing equivalents, namely NADH and flavin adenine dinucleotide (FADH2), which carry high-energy electrons. NADH and FADH2 donate electrons to the oxidative phosphorylation system, which is coupled with electron transfer along the mitochondrial respiratory chain, facilitating the production of energy in the form of ATP. This process leads to the heightened generation of free radicals [88]. The process of peroxisomal β-oxidation produces hydrogen peroxide and is not associated with phosphorylation mechanisms, as stated in reference [89,90]. The adaptive response triggered by lipid accumulation and redox balance disorder involves microsomal oxidation. Specifically, the cytochromes P4502E1 (CYP2E1) and P4504A are the primary microsomal contributors to oxidative stress in NAFLD. Consequently, NAFLD is characterized by an elevated rate of lipid catabolism within microsomes and peroxisomes, which is a contributing factor to OS [90,91].

### 3.3. NAFLD and Metabolic Diseases: Oxidative Stress and Antioxidant Markers

The escalation of oxidative stress, as previously documented, is a pathological state frequently observed in NAFLD. An imbalance such as that seen in NAFLD is also evident in several chronic illnesses, including arteriosclerosis, hypercholesterolemia [92], obesity [93], metabolic syndrome [94], and obstructive sleep apnea syndrome. These conditions are frequently linked to an elevated risk of cardiovascular disease. The overproduction of ROS is a causative factor in the oxidation of low-density lipoprotein (LDL), leading to the conversion of macrophages into foam cells. This process marks the initial stage of arteriosclerotic lesion formation. In contrast, the peroxidation of lipids in the liver by ROS has the potential to trigger inflammation and fibrosis through the activation of the hepatic stellate cell (HSC) compartment [95].

Limited research has been conducted to assess the antioxidant and pro-oxidant status in NAFLD. One of the most significant examples is a comprehensive collection of cases, which provides evidence of an elevated overall survival in vivo. The present study documented the effect through the assessment of urinary levels of 8-iso-prostaglandin F2α (8-iso-PGF2α), which is a byproduct of the non-enzymatic oxidation of arachidonic acid. They also measured the serum levels of soluble NOX2-derived peptide (sNOX2-dp), which serves as an indicator of NOX2 activation (i.e., the main NOX isoform responsible for ROS production). This investigation revealed that augmented concentrations of 8-iso-PGF2α and sNOX2-dp in individuals with NAFLD were not influenced by obesity, diabetes, or metabolic syndrome. Furthermore, these elevated levels were positively correlated with the severity of the disease as determined by liver ultrasound. Moreover, within the identical case series, the urinary concentrations of 8-iso-PGF2α exhibited an autonomous correlation with the serum concentrations of cytokeratin-18, which is an established hepatic indicator of apoptosis. This finding suggests a potential influence of liver impairment on systemic oxidative stress. A study conducted on a larger group of individuals with NAFLD revealed a noteworthy decrease in the levels of Vitamin E in their plasma. This decrease was observed in individuals with simple steatosis and in those with NASH, indicating the existence of heightened oxidative stress even in the initial stages of the disease. Vitamin E is a crucial fat-soluble antioxidant vitamin that safeguards cell membranes and lipoproteins from peroxidation, as a matter of fact. The circulating levels of a certain substance are regarded as a reliable indicator of antioxidant status and exhibit a negative correlation with markers of oxidative stress. One may postulate that the augmented OS observed in NAFLD could potentially lead to a depletion of endogenous antioxidants due to their surplus utilization [96].

**Figure 1:**
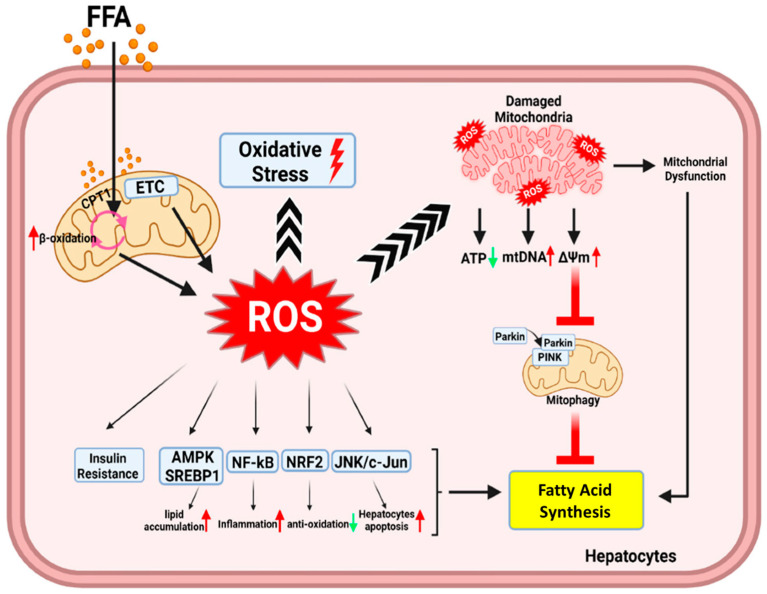
Schematic representation depicts how free fatty acids (FFA) enhance the generation of ROS by promoting lipid peroxidation and mitochondrial damage, which ultimately results in reduced ATP production and the initiation of mitophagy. Elevated levels of circulating FFA can lead to an excess influx of FFAs into hepatocytes. The excess FFAs undergo β-oxidation, generating ROS and causing damage to various cellular components, including mitochondria. Mitochondrial damage, in turn, leads to a reduction in ATP production. Additionally, the accumulation of damaged mitochondria stabilizes the mitophagy regulatory protein PTEN-induced protein kinase 1 (PINK1) and recruits the protein PARKIN from the cytosol. The PINK1-PARKIN protein complex facilitates the removal of damaged mitochondria through mitophagy. Reproduced with permission to modify from Yuanqiang Ma et al. [102].

Subsequent research has indicated that individuals with NAFLD display reduced brachial flow-mediated dilation, a recognized indicator of endothelial function and atherosclerosis. The outcome was notably conspicuous within the cohort of individuals diagnosed with NASH. Figure 1 explains the mechanism of ROS production via the free fatty acids (FFA) and how ROS generation reduces the efficiency of the mitochondria to produce energy. The underlying pathophysiological justification is that, in addition to low-grade systemic inflammation, OS is a significant contributor to the impairment of endothelial function, leading to a relative decrease in flow-mediated dilation. This hypothesis posits that the reduction of flow-mediated dilation is a significant predictor of cardiovascular events, as evidenced by numerous prospective studies [97,98]. To clarify, the rise in oxidative stress may be associated with the progression of NASH and cardiovascular ailments, indicating a potential correlation between these two disorders. Elevated levels of ROS have the potential to induce changes in platelet functionality and coagulation. Consequently, this leads to endothelial dysfunction and an elevated risk of thrombosis in various regions of the body, including the liver [99]. During the early stages of atherogenesis, the primary cause of damage to the endothelium is attributed to ROS and other pro-oxidant molecules. These molecules are produced by various cell types, including platelets, and are linked to the presence of risk factors associated with metabolic syndrome. The overproduction of ROS is causative of low-density lipoprotein (LDL) oxidation, leading to the conversion of macrophages into foamy cells. Similarly, ROS has the potential to stimulate the upregulation of scavenger receptors on smooth muscle cells, thereby facilitating their conversion into foam cells. Additionally, they have the ability to initiate alterations in the extracellular matrix through the stimulation of metalloproteinase secretion [100,101].

## 4. Nanoparticles—A Double-Edged Sword

Nanoparticles can indeed be considered a double-edged sword in the context of oxidative stress. On one hand, nanoparticles have shown potential in various applications, such as medicine, electronics, and environmental remediation. However, they can also induce oxidative stress and cause harm to living organisms including humans. When nanoparticles interact with biological systems, they can generate ROS through several mechanisms. For instance, certain nanoparticles such as metal-based nanoparticles (e.g., silver iron oxide) can directly produce ROS through redox reactions with cellular components [103]. Additionally, nanoparticles can induce oxidative stress indirectly by triggering inflammation, disrupting cellular antioxidant defenses, or interacting with mitochondrial function [104]. It is worth noting that the nanoparticles can also counteract oxidative stress rather than inducing it [105]. Nanoparticles can be engineered as antioxidant molecules in several ways. (i). Surface modification: The surface of the nanoparticles can be functionalized with antioxidant molecules or compounds. For example, nanoparticles can be coated or conjugated with natural antioxidants such as polyphenols, flavonoids, or vitamin E. (ii). Catalytic activity: Certain nanoparticles such as cerium oxide nanoparticles (nanoceria) possess an intrinsic catalytic activity that enables them to act as antioxidants. Nanoceria nanoparticles can mimic the activity of antioxidant enzymes such as SOD and CAT [106]. (iii). Free radicle scavenging: Nanoparticles such as fullerenes (e.g., C_6_O) have the ability to scavenge free radicles directly. Fullerenes can accept and stabilize unpaired electrons, naturalizing ROS and preventing their damaging effects. (iv). Nanoparticles composites: Nanoparticles can be incorporated into composite materials, where they act as antioxidants. For instance, nanoparticles can be embedded in polymers or other matrices, enhancing the overall antioxidant capacity of the material. Therefore, nanoparticles possess both pro- and antioxidant properties, and they hold promise for combating oxidative stress-related diseases. However, it is important to exercise caution due to their potential pro-oxidant activity as well.

The factors that are responsible for the pro-oxidant activity of nanoparticles include the nanoparticle’s active surface, size, photoactivation, toxins, dissolution of metal ions, and interactions with biomolecules. The impact of oxidative stress plays a significant role in the manifestation of nanotoxicity [104]. The entry of nanoparticles into cells and their subsequent release can result in the production of ROS within the cells. This, in turn, can cause an increase in ROS levels in the mitochondria, a decrease in ATP levels, and a disruption of the tricarboxylic acid (TCA) cycle [107,108]. Additionally, the lowered levels of cardiolipin, an essential phospholipid required for proper mitochondrial function, can lead to mitochondrial dysfunction. Significant factors that contribute to the generation of ROS induced by nanoparticles include the reactive surface of nanoparticle containing pro-oxidant functional groups, surface redox activation on NPs based on transition metals, and particle–cell interactions [109,110]. The antioxidant properties of metallic nanoparticles have been observed to exhibit enzyme-like behavior, leading to the scavenging of free radicals and subsequent reduction of ROS concentrations. Metal nanoparticles, including magnetic, silver, and gold nanoparticles, exhibit potential for the treatment and prevention of illnesses resulting from the overproduction of ROS. The creation of nanoparticles, also known as antioxidants, has been greatly improved through the integration of nanotechnology and materials science. The resulting nano-antioxidants substantially decrease the production of free radicals. Nano-antioxidants refer to nanoparticles that have been functionalized with either antioxidants or antioxidant enzymes or possess intrinsic antioxidant properties [111]. They are utilized as delivery systems for antioxidants. The properties that are considered to be of notable significance in terms of their antioxidant capacity comprise of activities such as superoxide dismutase, catalase, oxidase, and peroxidase-mimicking. Metallic nanomaterials (NMs) possess notable antioxidative properties owing to their capacity to transition between various oxidation states [112]. The precise mechanism underlying the antioxidant activity of nanoparticles remains elusive and requires further investigation. Additionally, the conflicting activity of nanoparticles as both oxidants and antioxidants can be attributable to their intricate interactions with biological systems. Whether nanoparticles exhibit oxidative or antioxidative properties depends on a number of variables. Additionally, the cellular context and the specific ROS involved play a critical role in determining the overall impact of nanoparticles on oxidative stress.

Ultimately, nanoparticles can act as a double-edged sword due to their ability to display both oxidant and antioxidant properties. While they can generate ROS and contribute to oxidative stress, they can also operate as antioxidants and alleviate the detrimental effects of ROS. Understanding the factors that govern nanoparticle behavior and their interactions with biological systems is crucial for harnessing their beneficial properties while minimizing potential risks. Nanomedicines, environmental remediation technologies, and other applications that make use of nanoparticles’ oxidant or antioxidant properties can benefit from careful engineering and regulation.

### 4.1. Nanoparticles as Pro-Oxidants

The continuous endogenous generation of ROS in a positive feedback cycle caused by metal nanoparticle-driven redox processes can lead to significant genotoxicity. The upregulation of inflammatory mediators such as nuclear factor kappa B (NF-κB), signal transducer and activator of transcription, mitochondrial dysfunction, and elevated intracellular calcium levels are what causes the chronic oxidative stress that develops after exposure to metal NPs [113]. They can produce reactive free radicals by either directly or indirectly activating the mitogen-activated protein kinase pathways [114,115]. As was previously mentioned, the physiochemical characteristics of metal nanoparticles, including their size, configuration, composition, shape, surface area, functionalization, charge, and magnetic properties, have a direct or indirect significant influence on the induction of oxidative stress and can result in genotoxicity or cellular toxicity [116]. When in the acidic lysosomal environment, NPs immediately contribute to the formation of ROS. NPs have been shown to alter gene expression and disrupt DNA strands [114]. According to a study, titanium oxide and other hydrophilic nanoparticles can cause ROS generation, which can promote the development of cancer. Titanium nanoparticles were discovered to cause mouse fibrosarcoma in one study, which subsequently developed into malignant tumors [115]. 

### 4.2. Nanoparticles as Antioxidants

**Figure 2:**
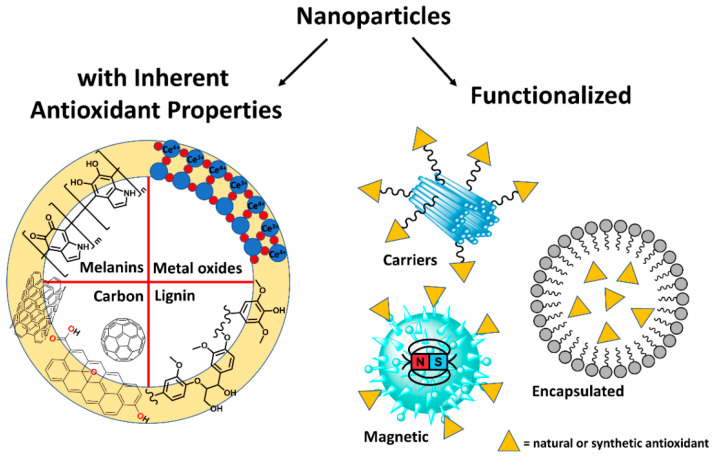
Schematic representation depicting the difference between functionalized antioxidant and inherent antioxidant ability exhibiting nanoparticles. Materials such as melanin and some metal oxides such as manganese and cerium oxides have the inherent ability to scavenge the existing free radicals, thereby reducing the ROS. Reproduced with permission from Andrea Baschieri and Riccardo Amorati [120].

The antioxidant properties of nanoparticles mainly depend on the method by which they are synthesized. There are many methods for preparation, including the solvent displacement method, supercritical fluid technology, emulsion or solvent evaporation, the templating technique, and the nanoprecipitation technique. New metal nanoparticles such as silver, gold, and the transition metal oxides of copper oxide and nickel oxide are widely used and researched for their antioxidant action. The antioxidant activity is increased by combining/incorporating different substances into single or bimetallic combinational NPs. Figure 2 explains the chemical makeup, nature, stability, surface-to-volume ratio, size, surface coating, and surface charge all play a role in antioxidant properties [117]. Due to their inherent physicochemical properties, some oxide nanoparticles can imitate antioxidant molecules or enzymes and scavenge reactive nitrogen and oxygen species [118]. A second method for quenching free radicals with nanoparticles relies on a nanomaterial’s capacity to do so by transforming alkyl peroxyl radicals to hydroperoxides [119].

## 5. Role of Nanoparticles in Scavenging the Free Radicals

Numerous studies have been conducted on natural, synthetic, and nanoparticle antioxidants and their potential in a wide range of applications. It includes gene delivery [121], theranostics for cardiovascular and neurodegenerative diseases [122,123], biomedical applications, and treatment for various toxicities caused by various environmental pollutants. To our knowledge, no research has been conducted on the types of nanoparticles and the integration of antioxidants to provide a comprehensive, up-to-date picture of this sector in a wider context. For effective and targeted distribution with sustained release features, we concentrate on the various methods for functionalizing nanoparticles, with antioxidants or molecules containing antioxidant properties in this review.

### 5.1. Role of Metal Nanoparticles as Antioxidants

#### 5.1.1. Silver Nanoparticles

The oxidant characteristics of silver nanoparticles that can prevent cell growth by interfering with membrane proteins or signaling pathways were covered in a previous section. Additionally, how silver nanoparticles can interact with protein sulfur groups was considered, particularly on antioxidant enzymes, and how they can hinder antioxidant action. The antioxidant properties of silver nanoparticles have been the subject of a great number of papers in recent years [111]. The science of oxidative stress is increasingly focusing on the use of nanoparticles as radical scavengers, for their redox potential, or as transporters for antioxidant chemicals [124]. The antioxidant qualities of silver nanoparticles (AgNP) may vary depending on how they are made, but in most cases, plant extracts are used to make them [125]. We can learn more about nanoforms and AgNPs’ antioxidant activities thanks to these phytochemicals. Brassica oleracea leaves were used by Ansar et al. [125,126] to create Ag NPs with good scavenging percentages ranging from 60 to 80%. The high antioxidant activity of these nanoparticles may be due to the quantity of phenolics and flavonoids that are produced on their surfaces as capping agents [125]. AgNPs from aerial Lavandula stoechas parts have been shown to scavenge DPPH radicals by 75% at a concentration of 25 mg/mL through the phytochemical components of phenols, terpenoids, and flavonoids. As AgNPs demonstrate how to exhibit both pro- and anti-oxidant effects based on their size and surface modification, a research group in 2022 found the size-dependent activity of the silver nanoparticles in inflamed liver tissues. In this research, two differently-sized AgNP were used in the treatment of LPS-contaminated liver slices alongside silymarin. The smaller-sized AgNP (10 and 75 nm) were combined with the larger-sized AgNP (250–300 nm) to achieve the desired effect. Biochemical studies revealed that both sizes of AgNP exhibited anti-inflammatory properties, but these properties were size-dependent. When compared to AgNps, large silver nanoparticles (AgNPL) considerably reduced LPS’s effects on TNF- and the proinflammatory mediator NO. However, for the proinflammatory cytokine IL-6, the effects of both AgNPL and AgNps were similarly significant. These results jived with prior accounts. The size of the AgNP particles utilized in experiments explains the variation in inflammatory mediator concentrations. The increased dispersion and toxicity of Ag in AgNps compared to AgNPL is related to the faster rate at which silver ion (Ag+) dissolution occurs in AgNps due to their greater surface area to volume ratio. This explains why NO and TNF- levels are so much higher in AgNps than in AgNPL. In vivo and in vitro studies have shown that hepatocytes respond to LPS, IL-1, TNF-, and reactive oxygen intermediates, all of which are known to favorably influence COX-2 production in other cell types. However, Kupffer cells and immortalized mouse liver cells retained the ability to express COX-2 but adult hepatocytes did not, despite the administration of pro-inflammatory stimuli. The production of PGH2 from arachidonic acid is the rate-limiting step in the synthesis of prostaglandins (PGs) and thromboxane, which COX-2 catalyzes. Intriguingly, hepatic COX-2 expression protects against acute liver damage by enhancing cell cycle progression and proliferation and decreasing apoptotic pathways in hepatocytes. In response to liver damage, the production of COX-2 increases anti-apoptotic genes and activates cell survival proteins, including phospho-Akt and phospho-AMP-kinase. However, these protective effects are lost when COX-2 is inhibited. In contrast to the AgNps group, the COX-2 expression was much higher in the AgNPL group. Their research verified the results of these other investigations and highlighted the important part played by AgNPLs [127]. As we come across this section, it can be found evident that, based on the size and the morphology, a nanomaterial can be tuned for its intrinsic functionality.

#### 5.1.2. Iron-Oxide Nanoparticles

The Fe_2_O_3_ NPs’ and Fe_3_O_4_ antioxidant properties have already been studied, and the theory behind them is based on the transfer of an electron to neutralize free radicals [128]. Nonetheless, it was effective in tailoring Fe2O3NPs using several methods, such as coating with carbon [129], carboxymethyl-inulin [130], and poly (GA), surface functionalization with natural antioxidant (GA) [131], and curcumin in magnetic–silk core-shell nanoparticles [132]. These customized Fe_2_O_3_NP composites displayed improved stability and dispersibility, and they were also assessed for their cytotoxicity and biocompatibility/hemocompatibility, as well as their effective antioxidant and antimicrobial properties and the ability to deliver drugs specifically to the target organs [129]. With average particle sizes of 5 and 8 nm, respectively, surface functionalized Fe_2_O_3_NPs with GA by in situ and post-synthesis showed 2–4-fold higher IC50 values in the DPPH antioxidant experiment than nonfunctionalized Fe_2_O_3_NPs. This improved free radical scavenging for Fe_2_O-NP@GA is caused by the synergistic action of Fe_2_O_3_-NP and GA. The free radical scavenging property is most likely due to electron transfer from Fe_2_O_3_-NP@GA to free radicals situated at the central nitrogen atom of DPPH. The antioxidant capacity of magnetite nanoparticles coated with GA-shell (PGA@MNPs), on the other hand, was tested in Jurkat cells in the presence of H_2_O_2_ as ROS, along with hemocompatibility and blood cell viability experiments. This coating was polymerized in situ at the surface of the particles in a soft and reagent-free process. Instead of showing any interaction with entire blood cells, PGA@MNPs significantly reduced the oxidative stress caused by H_2_O_2_. The in vitro assays showed that PGA@MNPs are both bioactive and biocompatible.

#### 5.1.3. Cerium Oxide Nanoparticles

The ability of cerium oxide nanoparticles (CNPs) to scavenge ROS/RNS and act as antioxidant enzyme mimics is largely dependent on the material’s inherent nanoscale physicochemical properties. In addition, it is influenced by the capacity to absorb and release oxygen and the relative thermodynamic efficiency of redox cycling between Ce^3+^ and Ce^4+^ ions on the surface of CNPs [133,134]. Moreover, CNPs have been successfully employed to treat a variety of malignancies, including the most recently targeted one, neuroblastoma, both in vitro and in vivo. However, the generation and accumulation of ROS with concurrent decreases in antioxidant enzyme levels are required for the anti-cancer properties of CNPs. The combination of CNPs with curcumin in a formulation may lead to improved physiological activity, since curcumin has anti-cancer capabilities. In a study, Kalashnikova et al. investigated the anticancer effects of curcumin-loaded nanoceria (CNP-Cur) and dextran-nanoceria (Dex-CNP-Cur) in neuroblastoma models using MYCN-amplified and non-amplified cell lines. In MYCN-amplified IMR-32 cells, Dex-CNP-Cur was found to cause significant cell death. It showed a 2-fold and 1.6-fold loss in cell viability for MYCN-upregulated and normal expressing cell lines, respectively, with little or very little toxicity in healthy cells (compared to untreated cells). Therefore, the dextran coating of CNPs not only aids in decreasing the survival of cancer cells but also aids in avoiding opsonization and phagocyte clearance of the nanoformulations from circulation. As a result, the formulation increases local curcumin concentration, stabilizes HIF-1, and upregulates caspase-dependent apoptosis, which in turn causes a long-term oxidative stress with CNP-assisted accumulation of ROS. CNP-Cur and Dex-CNP-Cur formulations cause neuroblastomas to produce more ROS and a significantly lower ratio of Bcl-2/Bax (Bax is an apoptosis-inducing gene and Bcl-2 stands for anti-apoptic factors), which leads to the release of cytochrome C and the activation of caspase 3/7 and apoptosis [134]. 

As a result of their antioxidant SOD- and CAT-mimetic activity, CNPs have shown that they can efficiently lower O_2_^•−^ and H_2_O_2_ levels. They have also shown that they are effective scavengers of ROS such as ^•^OH [135,136,137,138], and of RNS such as nitric oxide radical (^•^NO) [139,140] and peroxynitrite (O_2_NO] [141]. Das et al.’s study, which showed that CNPs were capable of removing ^•^OH generated from H_2_O_2_ in aqueous solutions, was one of the first to infer indirectly that they have inherent ^•^OH scavenging capability [136]. Later, based on NP size and Ce^3+^ surface levels, Xue et al. provided direct experimental proof that CNPs efficiently scavenge ^•^OH [137]. The CNPs became more efficient at scavenging ^•^OH and preventing a drop in the visible absorbance of methyl violet as the size of the CNPs decreased and as the level of Ce^3+^ on the surface of the NPs increased (higher Ce^3+^/Ce^4+^ surface ratios), according to a straightforward photometric study carried out by these authors. Another significant conclusion from this research was that the ROS scavenging activity of CNPs is significantly influenced by their capacity to flip reversibly from Ce^3+^ to Ce^4+^. Based on these findings, the authors proposed the following two-step mechanism for the ^•^OH scavenging activity of CNPs: Ce_2_O_3_ + 2[^•^OH] 2CeO_2_ + H_2_O (8) 2CeO_2_ (in presence of aqueous H^+^) Ce_2_O_3_ + 12O_2_. The first step indicates the oxidation of Ce^3+^ by ^•^OH and the second step indicates the reduction of Ce^4+^

Ce_2_O_3_ + 2[^•^OH] → 2CeO_2_ + H_2_O

2CeO_2_ (aqueous H+) → Ce_2_O_3_ + ½O_2_

Two recent studies demonstrate how CNPs can shield DNA from damage brought on by ^•^OH attack, adding more support for the antioxidant ^•^OH scavenging ability of CNPs [135,138]. The excessive synthesis of RNS, such as ^•^NO and O_2_NO, is known as nitrosative stress. Nitric oxide is not a particularly reactive chemical on its own [142]. However, when NO reacts with O_2_, it can create a wide range of dangerous species that are extremely reactive. When ^•^NO and O_2_^•− ^react, O_2_NO is formed. O_2_NO is a powerful oxidizing agent with a high potential for damage to lipids, proteins, and DNA, similar to the reactivity of ^•^OH. In two recent investigations, CNPs were proven to be an effective scavenger of ^•^NO [139,140]. In both experiments, CNPs with low Ce^3+^/Ce^4+^ surface ratios outperformed those with high Ce^3+^/Ce^4+^ surface ratios in terms of effectiveness. The following NO scavenging mechanism for the CNPs [140] was proposed by the authors:

Ce^4+^ + ^•^NO → [Ce^4+^ + NO ↔ Ce^3+^ + NO^+^]

Bernat Córdoba-Jover et al. introduced a novel approach for reducing ROS during liver regeneration through the utilization of nanoparticles. CeO_2_ nanomaterials offer several advantages over conventional anti-oxidative drugs. Firstly, they exhibit minimal toxicity even in cumulative doses. Secondly, CeO_2_NPs possess multi-enzyme mimetic activities that can effectively target various sources of ROS generation. Lastly, the catalytic activity of CeO_2_NPs can be continuously regenerated, thereby preventing the depletion of their anti-oxidative properties. In addition to the theoretical advantages over conventional drugs, their findings indicate that the therapeutic efficacy of the treatment under investigation surpassed that of the current standard of care, N-acetylcysteine, for managing acetaminophen toxicity in patients. The present study demonstrates that the administration of CeO_2_NPs is comparably efficacious in mitigating oxidative stress and tissue injury in rats subjected to APAP overdose, relative to N-acetylcysteine. Although NAC did not exhibit any impact on hepatocyte proliferation in damaged livers, CeO_2_NPs demonstrated a significant increase in cell proliferation both in vivo and in vitro. Furthermore, a noteworthy reduction in the proportion of HepG2 cells treated with CeO_2_NPs that underwent apoptosis was observed after 48 h of serum deprivation. This implies that the anti-apoptotic impact linked to the nanoceria treatment could potentially aid in augmenting liver regeneration. The transcription factor NF-κB is a significant contributor to the maintenance of liver homeostasis and the process of liver regeneration. As an illustration, mice with a knockout of NF-κB (p65) exhibit embryonic lethality and demonstrate extensive apoptosis of hepatocytes. Furthermore, the induction of hepatic IκB variants, which serve as inhibitors of NF-κB activity, prior to PHx, was correlated with hindered hepatic regeneration in rats. Moreover, the process of regeneration following partial hepatectomy was hindered when NF-κB was deactivated in both Kupffer cells and hepatocytes. In this study, it was demonstrated that the administration of CeO_2_NPs resulted in the activation of the transcription factor NF-κB both in vitro and in vivo. The current findings do not provide adequate evidence to establish a strong correlation between the activation of NF-κB and the advantageous outcomes of CeO_2_NPs therapy on the process of liver regeneration. Nevertheless, the concurrence between their discoveries and the aforementioned investigations renders this correlation feasible [143,144].

In a similar fashion, the activity of CeNPs were tested on rats with steatosis by Denise Oró et al. Rats treated with CCl_4_ and administered CeO_2_NPs exhibited distinct pathological characteristics compared to those administered with a vehicle. These include a significant reduction in liver fat accumulation and a lower incidence of portal hypertension. Hepatic fat accumulation is a consequence of heightened triglyceride synthesis within the hepatocytes. Irrespective of the etiology of intracellular lipid buildup in the hepatic tissue, augmented influx of free fatty acids leads to a state of mitochondrial β-oxidation overload, thereby elevating the burden on the endoplasmic reticulum. Dysfunction of the endoplasmic reticulum (ER) results in the generation of ROS, which triggers oxidative stress and initiates the inflammatory pathway. Furthermore, heightened levels of oxidative stress have been linked to hepatocellular apoptosis in rats exhibiting NASH induced by a high-fat diet. This occurrence appears to be facilitated by the activation of JNK and an inequity between pro- and anti-apoptotic proteins of the Bcl-2 family. The noteworthy aspect of this situation is the decrease in gene expression of Ncf1, Ncf2, Atf3, and Hspa5 that was observed following the administration of CeO_2_NPs to rats treated with CCl_4_. The Ncf1 and Ncf2 genes are responsible for encoding two subunits of NADPH oxidase, which is a complex enzyme utilized by cells for the generation of superoxide anions. In contrast, it has been observed that Atf3 and Hspa5 are molecules associated with endoplasmic reticulum stress that are modulated by ROS. Atf3 belongs to the family of transcription factors known as activation transcription factor (ATF)/cAMP responsive element binding (CREB), while Hspa5 encodes a member of the heat shock protein 70 family that participates in the process of protein folding and assembly within the endoplasmic reticulum. The aforementioned data suggest that the administration of CeO_2_NPs has the ability to impede oxidative and ROS-mediated ER stress in the context of liver injury induced by CCl_4_. Moreover, the significant decrease in TNFα, IL-1β, iNOS, and COX-2 expression observed in the liver of CeO_2_ nanoparticle-treated animals, as reported by D. Oro et al., supports the notion that the advantageous outcomes of these nanoparticles may be attributed to their potent antioxidant properties. The notion is reinforced by the restoration of PPARγ expression. It is established that the reduction in PPARγ expression prompts the activation of quiescent adipocytes, leading to complete differentiation into HSC. Additionally, PPARγ is indispensable in averting inflammation and preserving lipid and glucose homeostasis [145]. In reference to this context, the CNPs were found to be one of the top contenders as they exhibit a higher CAT activity and help in restoring the diseased liver. However, care should be taken in analyzing the complete toxicity of the nanoparticulate system.

#### 5.1.4. Manganese Oxide Nanoparticles

**Figure 3:**
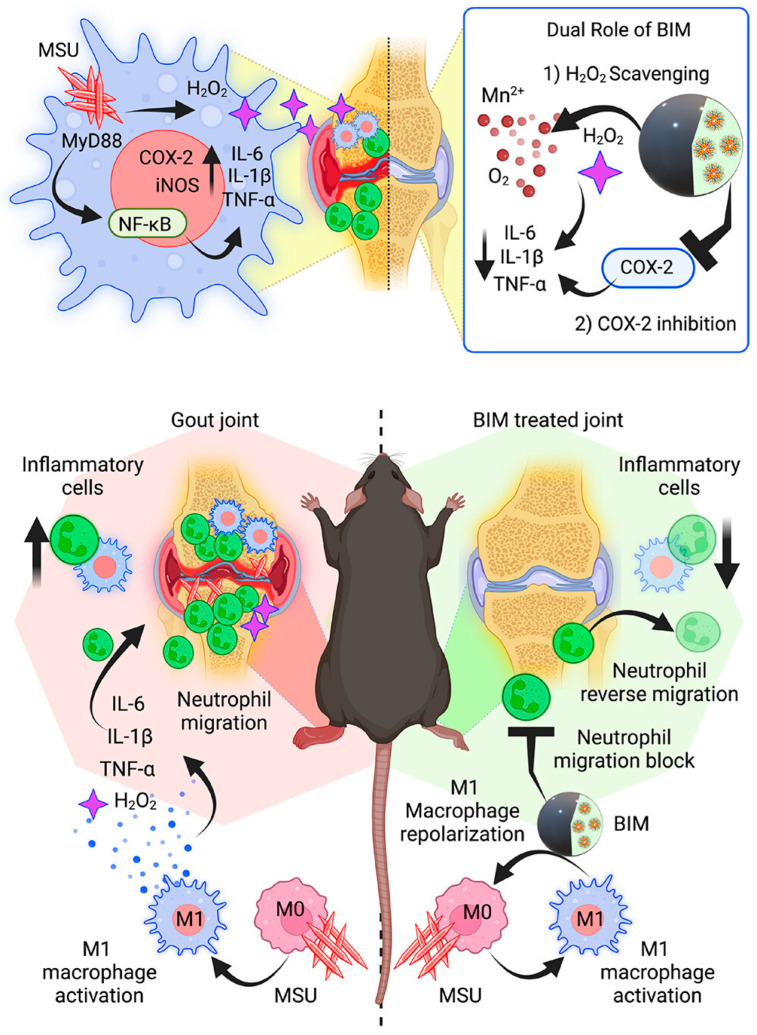
Schematic representation of the Manganese oxide nanoparticle in scavenging the excess ROS in gouty arthritis mice model. Adding to ROS scavenging the nanocomplex helps in the neutrophil reverse migration, thereby reducing the existing inflammation in the joint. The image is reproduced with permission from Adityanarayan Mohapatra et al. [28].

Manganese oxide was found to exhibit excellent anti-inflammatory activities via multiple pathways. On the other hand, MnNPs were also found to have an exceptional neutrophil reverse migration ability. Adityanarayan Mohapatra et al. prepared a biomineralized MnNp system and established its efficacy in treating the gouty arthritis mice model (in Figure 3). Here they have found a typical ability of MnNPs of clearing the existing neutrophils in the inflammatory site. They have proved the mechanism of neutrophil clearance in zebrafish model. Enough proofs were produced to showcase the nanoparticle’s ability to reduce the inflammation via iNOS, COX-2, and NF-κB pathways [28]. Similarly, Shreedevi Kumar et al. worked on PEGylated MnNPs for the protection of the cartilage from deterioration via inflammation-induced oxidative stress. The chondroprotective effects of the PEG-MnO_2_ NPs were determined in a cartilage explant model that mimicked OA, allowing for the detection of structural ECM degeneration and concurrent NO production, as well as in chondrocyte monolayers that were cytokine-challenged, allowing for the analysis of gene expression. Combined, these experiments enabled us to begin investigating potential free-radical scavenging NPs’ interactions with the cells’ overall oxidant-antioxidant systems. The results of both tests were consistent with one another in a number of respects. For instance, PEG-MnO_2_ NPs treatment lowered NO generation in cytokine-challenged cartilage explants and iNOS gene expression in cytokine-challenged chondrocytes. Furthermore, the biochemical results from the explant investigation, which showed that PEG-MnO_2_ NPs dramatically lowered release of GAGs in cytokine-challenged explants, are supported by the reduced expression of MMPs and ADAMTS genes by cytokine-challenged chondrocytes when treated with PEG-MnO_2_ NPs [146]. Recent research by Mengyun Peng et al. describes an effective activity of MnNPs in greater reduction of H_2_O_2_ via catalase mimicking activity. Their hypothesis was that hepatic hypoxia and oxidative stress stimulated HSCs continuously and chronically, making it difficult to reverse fibrosis. TGF-1 decreased CAT activity in the pro-fibrotic environment and then caused a buildup of H_2_O_2_. The increasing H_2_O_2_ in turn caused TGF-1 to rise even more, starting a vicious cycle. In order to break the circle, H_2_O_2_ was chosen as the target, and MnO_2_ was used to simulate CAT-like activity for H_2_O_2_ breakdown. By lowering hypoxia and oxidative stress, MnO_2_ altered the fibrotic milieu and decreased a pro-fibrotic stimulus from the source [147]. 

The cytokine TGF-β1 is commonly utilized to induce activation of HSCs and is considered a prototypical pro-fibrotic agent. Consequently, it has been extensively employed in the development of in vitro models of fibrosis. Previous studies have investigated the impact of TGF-β1 on CAT in airway smooth muscle cells, albeit with limited attention. A decrease in CAT activity has been documented in cases of liver and lung fibrosis for several decades. This reduction in CAT activity is believed to contribute to an imbalance in cellular redox. The findings of this study indicate that hepatic hypoxia leads to an upregulation of TGF-β1 expression, resulting in the inhibition of CAT expression in HSCs [148]. This inhibition is achieved through the downregulation of Foxo3a and Nrf2. The fibrotic area exhibited elevated levels of H_2_O_2_ accumulation and stabilized HIF-1α due to the inhibition of liver CAT. Additionally, the combined action of H_2_O_2_ and HIF-1α was found to induce an increase in the expression of TGF-β1, thereby establishing a detrimental feedback loop that poses a significant challenge for the treatment of liver fibrosis. The proposal posits that the hypoxic and oxidative stress conditions in the liver create persistent and long-term stimuli for HSCs, leading to challenges in the recovery of fibrosis. Within a pro-fibrotic milieu, TGF-β1 was observed to attenuate CAT activity, subsequently leading to an accumulation of H_2_O_2_. The surplus of hydrogen peroxide subsequently induced a further elevation of transforming growth factor beta 1 (TGF-β1), thereby establishing a detrimental feedback loop. Therefore, H_2_O_2_ was chosen as the focal point for disrupting the cycle, and MnO_2_ was utilized to emulate the catalase-like behavior for the decomposition of H_2_O_2_. The application of MnO_2_ resulted in the modification of the fibrotic microenvironment through the mitigation of hypoxia and oxidative stress, leading to a decrease in the pro-fibrotic stimulus originating from said environment [149]. The inhibition of CYP3A457 by Ssb1 and its deglycosylated metabolite were documented to augment liver targeting. Moreover, it has been observed that Saikosaponins, specifically Ssb1, exhibit hepatoprotective effects against liver injury induced by CCl_4_. As anticipated, the treatment of Ssb1 in isolation was observed to hinder the expression of α-SMA in in vitro trials. However, its efficacy in reversing liver fibrosis in Balb/c mice was limited. Conversely, MnO2@PLGA/Ssb1 demonstrated a more potent therapeutic effect in both in vitro and in vivo experiments. In brief, this study has established the significance of liver hypoxia and oxidative stress in the process of liver fibrogenesis. Additionally, we have identified that hypoxia-induced TGF-β1 plays a crucial role in regulating the expression of CAT in HSCs. The MnO_2_@PLGA/Ssb1 nanodrug demonstrated the ability to alleviate liver hypoxia and oxidative stress while also improving the antifibrotic efficacy of Ssb1. As such, it may serve as a viable therapeutic option for the treatment of liver fibrosis. This approach suggests that potentially efficacious remedies for hypoxia and OS could be implemented in various other fibrotic conditions, including but not limited to pulmonary fibrosis and renal fibrosis [147].

### 5.2. Role of Polymeric Nanoparticles as Antioxidants

One of the most promising nanocarriers being produced are polymeric nanoparticles, which are primarily made of synthetic biodegradable polymers. An aqueous extract of Syzygium cumini (ASc) seeds was combined with one of the well-known synthetic polymers recognized by the US-FDA, Poly (-caprolactone) (PCL), using an emulsification/evaporation solvent method. The DPPH radical scavenging ability and ferric reducing antioxidant power test (FRAP) were used to assess the ASc and PCL-ASc. According to the study, immobilization of ASc in PCL nanoparticle had no effect on antioxidant scavenging activity. Both ASc and PCL-ASc, even at a very low concentration (100 g/mL), have nearly the same and high scavenging DPPH radicals activity and reducing power in the FRAP assay [150]. Bacterial cellulose (BC), a biologically derived nanofiber-based polymer, has also attracted a lot of attention. This is mainly due to its superior film-forming capabilities, increased water-holding capacity, porosity, and—most importantly—biocompatibility. Silymarin and zein-containing spherical nanoparticles (SMN-Zein) can be adsorbed by BC, a nanocarrier. When SMN-Zein and BC films are combined, SMN-Zein/BC nanoparticles and nanofiber composites are created. SMN-Zein/BC have improved wettability, increased swelling of the BC films, increased solubility of sparingly soluble silymarin, and release from the nanocomposite films. In comparison to free SMN, SMN-Zein/BC demonstrated higher DPPH, ABTS%, and superoxide anion scavenging activity. Although BC lacked antioxidant activity, the composites’ antioxidant potential was increased by the gradual release of SMN because of its presence [151]. 

**Figure 4:**
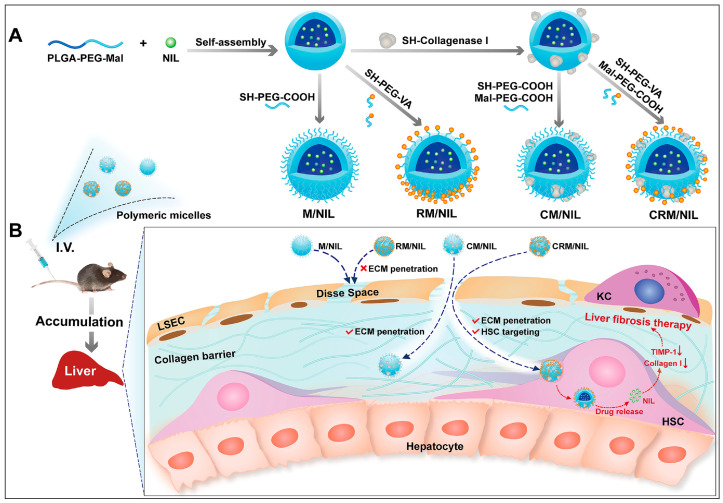
(**A**) Schematic representation depicting the process involved in the preparation of four distinct polymeric micelles. The surface of polymeric micelles was modified through a maleimide-thiol coupling reaction to attach collagenase I and retinol (also known as vitamin A or VA). (**B**) The figure depicts a schematic representation of the proposed fate of four distinct polymeric micelles within an in vivo setting. The CRM/NIL, which resembles a nanodrill, exhibits the capability to infiltrate the collagen barrier and selectively targets activated HSCs. The process of internalizing CRM/NIL results in the liberation of NIL, leading to a decrease in the expression of TIMP-1, a metallopeptidase inhibitor. This subsequently promotes the degradation of collagen I, ultimately exhibiting a therapeutic effect against liver fibrosis. Reproduced with permission from Qian-Qian Fan et al. [152].

The efficacy of nano-drug delivery system targeting HSCs was impeded to a significant extent due to the excessive accumulation of fibrosis collagen in the space of Disse, which is associated with hepatic fibrogenesis. The present investigation involved the development of a polymeric micelle (CRM) with a nanodrill-like structure. The study demonstrated the ability of the CRM to effectively penetrate the collagen barrier that is typically present in fibrotic liver and achieve optimal targeting of HSCs. Concurrently, we also fabricated three additional unadorned polymeric micelles (M, RM, and CM) for the purpose of comparison. Similar physicochemical properties were observed among the four distinct polymeric micelles. The polymeric micelles were evaluated for their cellular uptake in the context of an excessive collagen I barrier. Among the four types of micelles, the nanodrill-like CRM exhibited the highest cellular uptake. This can be attributed to the proteolysis function of collagenase I and the enhanced-uptake effect of the ligand retinol that decorates the CRM. Furthermore, Confocal Laser Scanning Microscopy (CLSM) demonstrated that the Carrier-Mediated Transport (CMT) agent efficiently discharged intracellular cargo within LX-2 cells. A two-stage, non-fatal hepatic fibrosis model was established through the administration of CCl4 via intraperitoneal injection over a period of 4 or 8 weeks. This model was utilized to evaluate the liver’s stage-dependent accumulation of polymeric micelles. In Figure 4, the findings of this study indicate that in mice subjected to an 8-week CCl_4_ treatment, CRM demonstrated the most significant accumulation in the liver. However, in mice exposed to a 4-week CCl_4_ treatment, both CRM and CM exhibited comparably high levels of accumulation in the liver. Moreover, the utilization of immunofluorescence staining revealed that the delivery of cargos to activated HSC was more effective with the use of cell-penetrating peptides conjugated with arginine-rich motifs in comparison to cell-penetrating peptides conjugated with membrane translocation domains (CM). Furthermore, the utilization of CRM in conjunction with the antifibrotic agent NIL resulted in the most favorable antifibrotic outcomes in the nonfatal hepatic fibrosis model, which was induced by sequential intraperitoneal administration of CCl4 for a duration of 8 weeks. This approach exhibited a substantial accumulation of the drug in the liver and effective targeting of HSC. Significantly, it was demonstrated that CRM displays exceptional cell compatibility and hemocompatibility in vitro and does not manifest any acute or chronic toxicity in vivo. Based on the aforementioned results, it is suggested that utilizing CRM as a nanodrug delivery system could be a promising approach for targeting HSCs in the treatment of liver fibrosis. In addition, our study demonstrates the novel finding that surface functionalization of a nanocarrier with collagenase I enhances its ability to penetrate the fibrotic liver and achieve favorable accumulation. This finding suggests that the utilization of collagenase I modification may serve as a novel approach in the development of more effective carriers for the precise treatment of liver fibrosis [152]. As a conclusion, polymeric nanoparticles are a better option in targeting specific types of cells in liver by conjugating targeting peptide or antibody to it. It is also a safer option in delivering a combination of drugs via a single system.

## 6. Future Prospective

Nanoparticles have emerged as promising candidates for the treatment of oxidative stress-related liver disorders, owing to their unique physicochemical properties and versatile therapeutic capabilities. These tiny particles possess a high surface-to-volume ratio, allowing for efficient drug loading and delivery to the affected liver tissues. Moreover, nanoparticles can be engineered to exhibit antioxidant properties, which counteract the harmful effects of oxidative stress on the liver. By encapsulating potent antioxidants within nanoparticles, such as polyphenols or vitamins, their stability and bioavailability can be significantly enhanced. These nanoparticles can selectively target the liver, either through passive accumulation or active targeting strategies, thereby minimizing off-target effects and maximizing therapeutic efficacy. Furthermore, nanoparticles can protect the encapsulated antioxidants from degradation, ensuring their sustained release within the liver and providing a prolonged therapeutic effect. The use of nanoparticles as a drug delivery system holds immense potential for combating oxidative stress-related liver disorders, offering a novel approach for precise and effective treatment.

## 7. Limitations 

Nanoparticle systems have encountered challenges in gaining entry into the clinical market for a considerable duration, especially for treating oxidative stress-related hepatic diseases. This phenomenon can be attributed to the absence of precise characteristics and significant levels of toxicity. Over the course of several years, research in the field of nanotechnology has undergone a significant transformation, evolving from a relatively unknown area of study to a promising avenue for therapeutic applications. However, the persistence of this phenomenon within the realm of liver treatment remains an unresolved enigma. While nanotechnology presents significant potential in addressing oxidative stress-related liver disorders, it is essential to address the safety and biocompatibility of nanomaterials. Thorough evaluation of nanoparticle toxicity, biodegradability, and potential accumulation in the liver is crucial to ensure their safe clinical translation. The design of the nanoformulation should prioritize long-term efficacy while also ensuring that the formulation does not elicit hepatotoxicity, which could potentially exacerbate liver injury. It is imperative to bear in mind these limitations as we transition into the age of nanotechnology for diverse therapeutic applications.

## 8. Conclusions

The present review pertains to the examination of the oxidant and antioxidant characteristics of nanoparticles for their potential application in the treatment of liver diseases. Despite the limited research on the application of nanoparticles in treating liver diseases, our discussion has focused on the utilization of nanoparticles in treating hepatic steatosis and the specific mechanism of action of nanoparticles. By enabling targeted antioxidant delivery, mitochondria protection, ROS scavenging, and enhanced diagnostics, nanotechnology offers innovative solutions to combat oxidative stress-related liver diseases effectively. Further research and development are warranted to optimize nanosystems, ensure their safety, and translate these advancements into clinical practice. With continued progress, nanotechnology has the potential to revolutionize the treatment landscape for oxidative stress-related liver disorders, ultimately improving patient outcomes and quality of life.
